# Evaluation of six decontamination procedures for isolation of *Mycobacterium avium* complex from avian feces

**DOI:** 10.1371/journal.pone.0202034

**Published:** 2018-08-10

**Authors:** Abdul Sattar, Zunita Zakaria, Jalila Abu, Saleha A. Aziz, Rojas-Ponce Gabriel

**Affiliations:** 1 Department of Veterinary Pathology and Microbiology, Faculty of Veterinary Medicine, Universiti Putra Malaysia UPM, Selangor, Malaysia; 2 Department of Clinical Studies, Faculty of Veterinary Medicine, Universiti Putra Malaysia UPM, Selangor, Malaysia; 3 Department of Electrical and Computer Engineering, University of Alberta, Edmonton, Canada; University of Helsinki, FINLAND

## Abstract

Culture is considered the gold standard for definitive diagnosis of mycobacterial infections. However, consensus about the most suitable culture procedure for isolation of nontuberculous mycobacteria is lacking. The study compared the recoveries of mycobacteria after decontamination of spiked and fresh avian feces with 4% sodium hydroxide (NaOH), 12% sulfuric acid (H_2_SO_4_), or 1% cetylperidinium chloride (CPC), with and without mixture of three antibiotics, namely vancomycin (VAN, 100 μg/ml), nalidixic acid (NAL, 100 μg/ml), and amphotericin B (AMB, 100 μg/ml). The antibiotic mixture was referred to as VNA. Decontamination procedures were evaluated using two (*n* = 2) avian fecal samples spiked with 10^6^, 10^4^, and 10^2^ CFU/ml of *Mycobacterium avium* subsp. *avium* (ATCC 15769) and fresh avian feces (*n* = 42). *M*. *avium* subsp. *avium* was detected on the culture media from spiked samples (10^6^ and 10^4^ CFU/ml) decontaminated with NaOH, NaOH-VNA, H_2_SO_4_, and H_2_SO_4_ -VNA for 2−6 weeks. These bacteria were detected in 2–4 weeks when using CPC and CPC-VNA. *M*. *avium* subsp. *avium* cannot be isolated on culture media from spiked samples (10^2^ CFU/ml) decontaminated with any decontaminating agent. Two mycobacterial isolates, namely, *Mycobacterium terrae* and *M*. *engbaekii*, were isolated from field samples decontaminated with NaOH and CPC-VNA. With regard to the contamination rate, the use of CPC-VNA showed lower contamination rates (5.5% and 19.0%) from spiked and field samples than those of the other methods (NaOH: 22.2% and 59.5%, NaOH-VNA: 16.7% and 21.4%, H_2_SO_4_: 11.1% and 40.5%, H_2_SO_4_-VNA: 5.5% and 21.4%, and CPC: 66.7% and 50%). In conclusion, the decontamination of fecal samples following a two-step procedure with 1% CPC and VNA can ensure high recovery rate of many mycobacteria with the lowest contamination in cultures.

## Introduction

*Mycobacterium avium* complex (MAC) includes two closely related species, namely, *M*. *avium* and *M*. *intracellulare*, which are saprophytes and opportunistic pathogens to human and animals [1]. MAC causes chronic gastrointestinal infection in almost all bird species [1] and significant loss to rare and endangered avian species in zoo and breeding establishments [1]. MAC is an emerging pathogen with considerable public health importance [2,3] because it can cause severe disseminated diseases in patients immunocompromised due to AIDS, cystic fibrosis, leukemia [4,5,6], and pulmonary disease in immunocompetent people [4,7,8]. Centers of Disease Control and Prevention reported that the median annual incidence of nontuberculous mycobacteria (NTM) is 110/100000 HIV-positive persons/year during 2007–2012, and MAC is frequently isolated among NTMs [9]. *M*. *intracellulare* causes 40% respiratory disease in immunocompetent patients with chronic lung disease [4]. For human exposure to MAC, birds are important agents to spread MAC [10]. Birds may cause environmental contamination with MAC through fecal droppings, thereby increasing public health concern [1]. Immunocompromised patients may contract the pathogen during handling of infected birds or consumption of meat from infected birds [11,12].

Diagnosis of avian mycobacteriosis is commonly made by pathological findings at postmortem and isolation [13]. In human, diagnosis of disseminated MAC infection is performed by biopsy of lymph node, liver and bone marrow. Diagnosis of pulmonary infection is performed by radiology of chest, acid fast staining and culture of sputum [14]. Antemortem in live birds is challenging [15] because most birds show no clinical signs of the disease [1]. Infected birds excrete mycobacteria through their feces, depending on the stage of the disease. Mycobacterial shedding through feces is high in advanced stage of the disease [13]. Feces have successfully been evaluated for screening of mycobacterial infections in ruminants [16]. Avian feces may be a suitable non-invasive source for diagnosis of avian mycobacteriosis [13].

Fecal culture is considered the gold standard for diagnosis of mycobacteriosis in live animals [16]. However, culture is time consuming because mycobacteria pathogenic to birds are slow growing and colonies may take 2 to 4 weeks to appear due to long replication time of at least 15 h [13]. Molecular techniques like polymerase chain reaction (PCR) and genotyping and sequencing of *16S rRNA* and *hsp*16 gene have revolutionized the detection of NTM in human and animal samples [17,18,19]. Molecular methods are rapid and result can be available in one day [17]. However, cultivation and isolation of pathogen is essential for subsequent identification and antimicrobial susceptibility test [16]. Culture can detect most animals in advanced stage of infection [13]. However, given that the isolation of mycobacteria on culture media from fecal culture is a challenging task due to the complex microbiota in the gut [15], which can mask the growth of mycobacteria, its application is discouraged [16]. Although several culture procedures have been developed for mycobacterial cultivation [20,21], consensus on a single culture procedure for NTM due to the presence of contaminating organisms in feces and long incubation period of mycobacteria is lacking [22]. The most used chemicals for decontamination of fecal samples are sodium hydroxide (NaOH), hydrochloric acid (HCl), sulfuric acid (H_2_SO_4_), oxalic acid, cetylperidinium chloride (CPC), benzalkonium chloride, trisodium phosphate, and sodium chloride [13,14]. All these chemicals exert adverse effects on the growth of mycobacteria [21]. Chemical effects should be balanced to eliminate contaminating microorganisms and to support mycobacterial growth [21]. A number of previous studies have recommended that CPC is the most suitable chemical [23,24]. Similarly, the low toxicity of CPC to mycobacteria enables a fast recovery rate of mycobacteria [21,24].

Chemical decontamination is insufficient to eliminate all nontarget organisms [16]. Incorporation of antimicrobials in the decontamination procedure will remove most of the contaminant bacteria and provide opportunity for bacilli to grow, which results in highly positive cultures [11,16]. The use of antibiotics, such as vancomycin (VAN), nalidixic acid (NAL), and amphotericin B (AMB), in previous studies has shown desired effects by improving culture sensitivity and reducing contamination rate [16]. Nonetheless, these antibiotics present inhibitory effects on the growth of mycobacteria [13,16]. Minor inhibitory effects can be ignored because of significant improvement in the sensitivity of culture due to the use of antibiotics [16].

Given that the decontamination method of fecal samples is one of the main factors affecting the isolation of MAC from culture media, the present study aimed to evaluate three decontaminating agents, namely, 4% NaOH, 12% H_2_SO_4_, and 1% CPC, with the presence or absence of VAN, NAL, and AMB (VNA) antibiotics to recover MAC on Löwenstein–Jensen (L–J) culture medium from spiked fecal samples and fresh avian feces.

## Materials and methods

### Preparation of decontaminating agents

Decontaminating agents, such as 4% NaOH, 12% H_2_SO_4_, and 1% CPC, were prepared by dissolving 20 g of NaOH pellets in 500 ml of distilled water (4% NaOH). A total of 60 ml of 98% H_2_SO_4_ was added to 440 ml of distilled water (12% H_2_SO_4_). Furthermore, 5 g of CPC and 10 g of sodium chloride were dissolved in 500 ml of distilled water (1% CPC). All three solutions were sterilized by autoclaving at 121 °C for 15 min. NaOH and H_2_SO_4_ solutions were stored at room temperature, and CPC solution was kept in the dark at room temperature. NaOH and H_2_SO_4_ solutions remain stable at room temperature, whereas CPC solution is degraded by exposure to light, extreme temperature, and evaporation [25].

### Preparation of antibiotic stock solutions

Antibiotic (10 mg/ml VAN, 10 mg/ml NAL, and 5 mg/ml AMB) stocks were prepared. VAN (20 mg) was dissolved in 2 ml of sterile distilled water. NAL (20 mg) was dissolved in 1.5 ml of sterile distilled water and completely dissolved by adding 0.5 ml of 2% NaOH. AMB (10 mg) was solubilized in 1.5 ml of dimethyl sulfoxide, and the final volume of 2 ml was obtained by adding 0.5 ml of sterile distilled water. The solutions were filtered using Millex HA filter unit (0.45 μm) syringe and stored at −20 °C. Antibiotic working mixture was prepared by adding 0.5 ml of each antibiotic stock of VAN, AMB, and NAL to 48.5 ml of sterilized distilled water. In the working antibiotic mixture, the final concentration of VAN and NAL was 100 μg/m and that of AMB was 50 μg/ml. Working antibiotic mixture was aliquoted in sterile snap cap microtube (2 ml). Antibiotic working mixture was referred to as VNA (VAN, NAL, and AMB).

### Preparation of Löwenstein Jensen media

Löwenstein Jensen (L–J) media were prepared according to the manufacturer’s instruction with slight modification in the coagulation step. The prepared L–J medium solution (8–10 ml) was dispensed in universal bijou bottles (28 ml), subsequently arranged in slanting position in a stainless steel tray, and incubated in a preheated hot air oven at 90 °C for 90 min instead of 85 °C for 45 min in an inspissator. The time and temperature combination was optimized in this study. After coagulation, the slants were allowed to cool at room temperature overnight. In the current study, only L-J was used for cultivation of mycobacteria because CPC along with other decontaminating agents was used for decontamination of feces. CPC is compatible with L-J. Its bacteriostatic effects are completely neutralized on L-J. CPC is not compatible with agars, growth of mycobacteria on agar is inhibited due to bacteriostatic effects of CPC [26,27].

### Preparation of standard concentrations of *Mycobacterium avium* subsp. *avium*

*M*. *avium* subsp. *avium* Chester (ATCC 15769) isolates, which were provided by the Bacteriology Laboratory, Faculty of Veterinary Medicine, Universiti Putra Malaysia, were grown on L–J slants at 37 °C. *M*. *avium* subsp. *avium* Chester (ATCC 15769) belongs to serotype 1, which is virulent to all avian species. After sufficient growth, all colonies were transferred to sterile centrifuge tube (15 ml), which contained 5 ml of sterile phosphate buffer solution (PBS) with a pH level of 6.8 and five sterile glass beads (3 mm). The suspension was homogenized on vortex for 1 min. To settle large clumps of mycobacterial colonies, the suspension was allowed to stand undisturbed for 30 min at room temperature. The supernatant was transferred to a new sterile tube. The turbidity of the suspension was adjusted to McFarland 0.5 standard (1.5 x 10^8^ CFU/ml) [28]. Tenfold serial dilutions of the suspension adjusted to McFarland 0.5 were prepared using sterile distilled water. Using of visual inspection of suspension turbidity rather than actual plated CFU counts, is a limitation of this study. Three serially diluted suspensions containing approximately 10^7^, 10^5^, and 10^3^ CFU/ml were selected to spike fecal samples. These inocula were selected to cover the range of number of organisms that will be present in the clinical samples [29]. Inoculum (10^5^ CFU/ml) and sterile distilled water were used as positive and negative controls, respectively. Positive and negative controls were unexposed to chemical decontamination.

### Preparation of fecal samples

Three replicates (0.2 g) were prepared for each sample. Briefly, 0.2 g of faecal sample was transferred to a 15 ml sterile polyethylene tube containing 10 ml of sterile distilled water. The sample was mixed and homogenized by vortexing for 1 min at 500 rpm. The tubes were then allowed to stand for 30 min at room temperature before filtration of supernatant through sterile surgical gauze (mesh size, 19x15). Filtration helps to prevent carryover of large fiber particles that may cause contamination of samples. The filtrates were subjected to spiking with *M*. *avium* subsp. *avium* (ATCC 15769).

### Spiking fecal samples with *M*. *avium* subsp. *avium*

Three filtrates of each fecal sample (approximately 9 ml in a 15 ml tube) were inoculated with 1 ml of *M*. *avium* subsp. *avium* suspensions with initial concentrations of 10^7^, 10^5^, and 10^3^ CFU/ml. The suspensions were mixed well on the vortex and centrifuged at 3000 x*g* using refrigerated centrifuge. The supernatants were discarded, and pellets were resuspended in 3 ml of sterile distilled water, mixed on vortex, and subdivided into three aliquots. The estimated numbers of *M*. *avium* subsp. *avium* in each aliquot, which was subjected to decontamination, were approximately 10^6^, 10^4^, and 10^2^ CFU/ml. To minimize the loss of bacilli during the first sedimentation and filtration, feces were spiked after filtration. After spiking of fecal filtrates, each filtrate was divided into three aliquots for decontamination with three decontaminating agents, which reduced the number of organism by threefold.

### Decontamination of fecal samples

**Fig 1:**
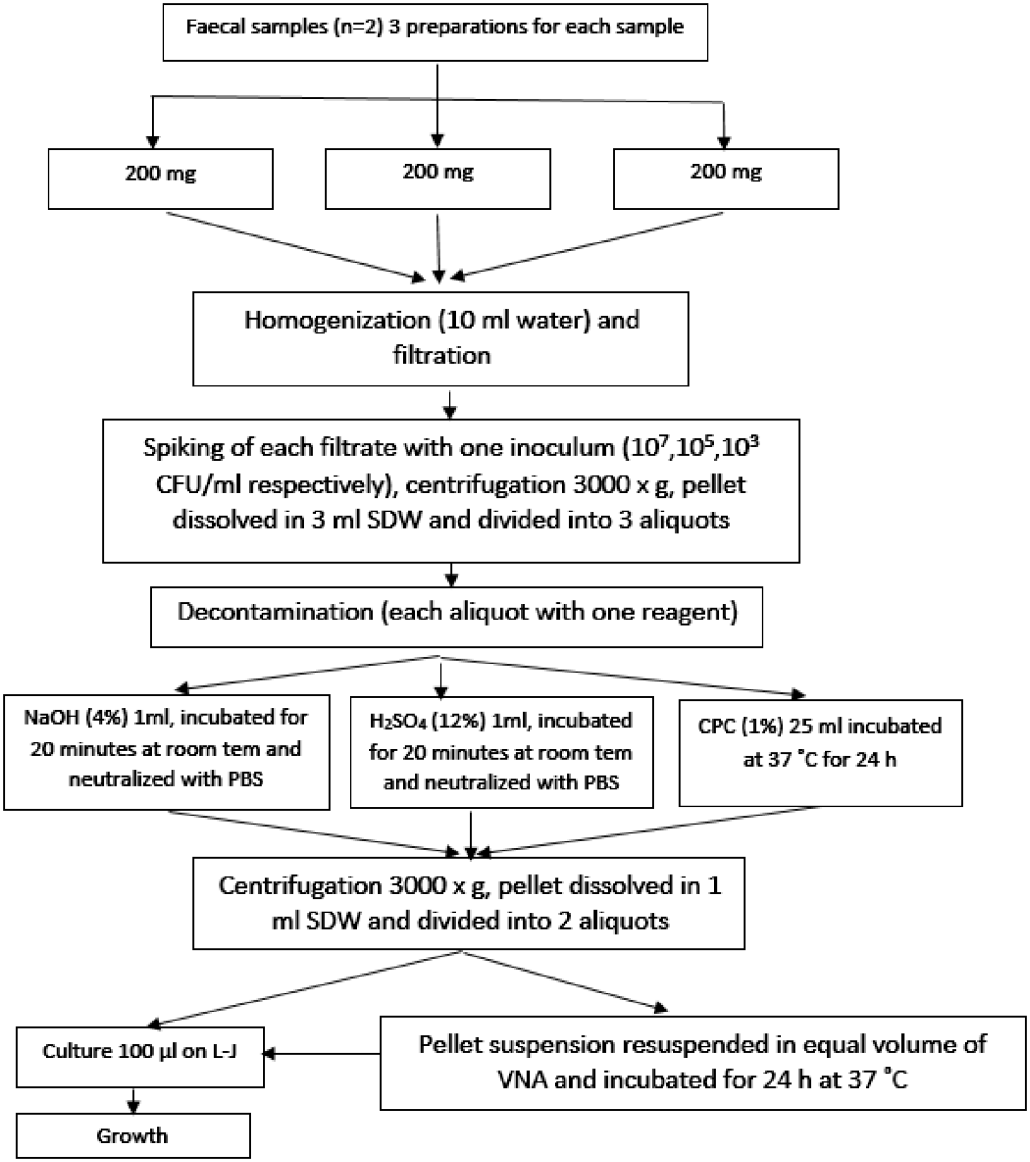
Schematic illustration for samples preparation, spiking with *M*. *avium subsp*. *avium* and decontamination with six procedures.

Each of the three aliquots of a pellet sediment (1 ml) was transferred to a 50 ml Falcon tube and decontaminated with 4% NaOH, 12% H_2_SO_4_, and 1% CPC [22]. The first and second aliquots were mixed with equal volume of 4% NaOH (2% final concentration) and 12% H_2_SO_4_ (6% final concentration), respectively. The third aliquot was mixed with 25 ml of 1% CPC. The suspensions were mixed well by inverting the tubes and vortexing at 500 rpm for 30 s. The inner surface of the Falcon tubes, including the inner surface of the cap, was exposed to decontaminating agents to minimize the possibility of contamination. Pellet aliquots decontaminated with NaOH and H_2_SO_4_ were allowed to stand for 20 min at room temperature, and those aliquots decontaminated with CPC were kept at 37 °C for 24 h. After decontamination for 20 min, NaOH and H_2_SO_4_ activities were neutralized with 48 ml of PBS (pH = 6.8) [30]. The final pH of the suspension was determined by using pH strip and stabilized between 6.5 and 7.0 [30]. All centrifugations were performed at 3000 x*g* for 15 min. Aliquots decontaminated with NaOH and H_2_SO_4_ were centrifuged at 4 °C, and those treated with 1% CPC were centrifuged at 10 °C [31,32]. Supernatants were discarded, and pellets were resuspended in 1 ml of sterile distilled water. A 100 μL of pellet sediment was inoculated onto L–J slant in triplicate. The remaining pellet sediment (600 μl) was resuspended with equal volume of VNA, mixed by vortexing at 500 rpm for 30 s, and incubated at 37 °C for 24 h. Finally, 100 μL of pellet sediment treated with VNA was inoculated onto L–J slant in triplicate (Fig 1).

### Isolation of mycobacteria from fresh avian feces

#### Collection of fecal sample

Fecal samples from exotic birds (*n* = 7) and village chickens (*n* = 35) were collected from May to October 2016. Exotic birds included three indoor wild birds [common kestrel (*Falco tinnunculus*, *n* = 2) and owl (*Tyto alba*, *n* = 1)) and four pet birds (macaw (*Ara* spp., *n* = 1), amazon parrot (*Amazona* spp., *n* = 2), and green checked parrot (*Pyrrhura molinae*, *n* = 1)]. These birds were either kept as indoor patients or brought for treatment to the Universiti Veterinary Hospital, Faculty of Veterinary Medicine (Lat.2.975634-Lng.101.711232) Universiti Putra Malaysia. Village chickens (*Gallus domesticus*) were sampled from a private poultry farm in Puchong (Lat.2.962213-Lng.101.634525) and backyard domestic chickens in Seri Serdang (Lat.3.010499-Lng.101.706776), Selangor, Malaysia. No study has been published about the occurrence of *M*. *avium* in the study area. Clean newspapers or plastic sheets were placed beneath cages or near the water and feeding troughs, and the birds were monitored. Fecal samples were collected immediately in a new 50 ml Falcon tube after the birds deposited feces. Falcon tubes containing samples were placed in an insulated container with ice packs, transported, and processed in the Bacteriology Laboratory, Faculty of Veterinary Medicine, Universiti Putra, Malaysia. Samples that were unprocessed the same day due to work load were stored at 4 °C for not more than 24 h. No specific permission was required for fecal collection because this study involved no bird manipulation. Fecal samples were collected after the birds deposited feces. The land was privately owned. This study involved no endangered or protected species.

#### Preparation of fresh fecal samples

**Fig 2:**
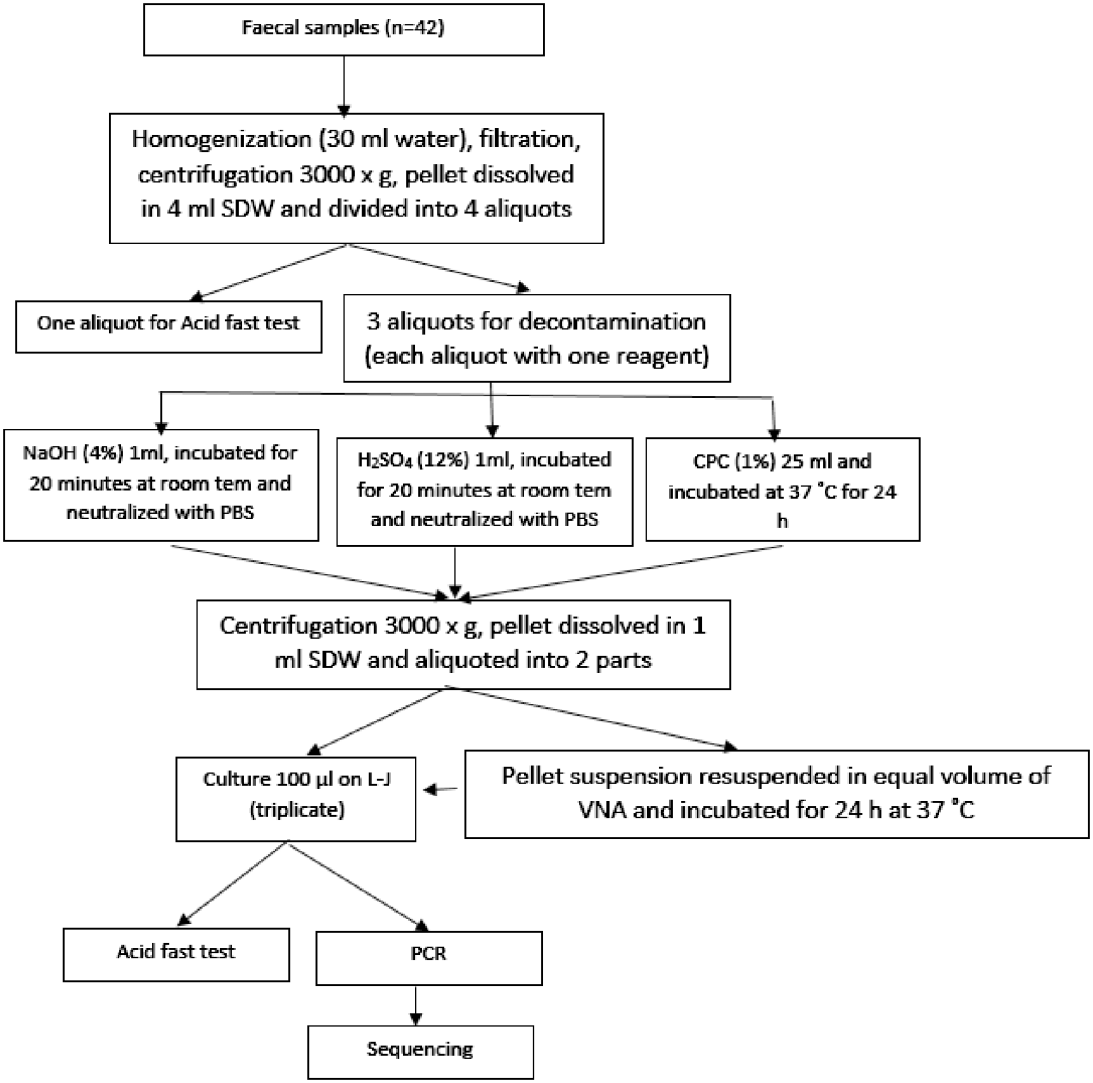
Schematic illustration for fresh fecal samples preparation and decontamination with six procedures.

Fresh feces (1 g) were dissolved in 30 ml of sterile distilled water in a 50 ml Falcon tube. Homogenization, filtration, and centrifugation protocols were same as described previously (Fig 2). The supernatant was poured out, and the pellet was resuspended in 4 ml of sterile distilled water and divided into four aliquots. Three aliquots were subjected to decontamination procedures as described previously. The fourth aliquot was used for acid fast smear preparation. Smears were stained with Ziehl–Neelsen stain and examined using 100× oil immersion magnification.

#### Culture of fecal samples on Löwenstein Jensen media

Löwenstein Jensen slants were incubated at 37 °C for 8 weeks before reporting the final results. L–J slant cultures were kept in slanted positions with loose caps for one week and examined daily for any contamination. After the first week of incubation, the cultures were placed in upright position, and caps were tightened. Cultures were examined weekly for mycobacterial growth. All growths were evaluated for acid fast bacilli (AFB). Colonies were dissolved in a drop of normal saline and spread on a clean glass slide. The smears were air dried and heat fixed by passing the glass slide over the burner flame to ensure proper adherence. The smears were stained with Ziehl-Neelsen stain and examined using 100× oil immersion magnification. In acid fast smears, mycobacteria appeared as red rods in chains and clumped with a blue back ground. AFB-negative cultures were considered contaminations.

### Molecular speciation of isolates grown on L–J media

#### DNA extraction

DNA was extracted from mycobacterial colonies grown on L–J slants using blood and tissue kit (Qiagen^®^) according to the manufacturer’s instruction, with several modifications on the quantity of elution buffer to obtain high DNA concentration. Briefly, a loop full of fresh colonies was dissolved in 200 μL of lysis buffer and incubated at 37 °C for 1 h in a dry heat block. After incubation, 20 μL of proteinase K and 200 μL of AL buffer were added to the bacterial suspension, mixed well on a vortex, and incubated for 10 min at 70 °C. Afterward, 200 μL of 96% ethanol was added to the suspension and mixed well. The suspension was transferred into the DNeasy Mini-column and centrifuged for 1 min at 5900 x*g*. DNA was washed two times with washing buffers. DNA was first washed with 500 μL of AW1 buffer and centrifuged for 1 min at 15600 x*g*. In the second washing, 500 μL of AW2 buffer was used, and DNA was centrifuged for 3 min at 15600 x*g*. Finally, the DNeasy Mini-column was transferred to 1.5 ml PCR tubes, and DNA was eluded with 50 μL of AE buffer. After incubation for 1 min at room temperature, DNeasy Mini-column was centrifuged for 1 min at 5900 x*g*. The quality of eluted DNA was assessed by gel electrophoresis using 0.8% agarose gel. DNA was stored at −20 °C.

#### PCR amplification

PCR amplification was carried out using TopTaq^™^ Master Mix (Qiagen^®^) according to the manufacturer’s instruction. Briefly, each 25 μL of reaction mixture contained 12.5 μL of TopTaq Master Mix 2x, 6.5 μL of RNase-free water, 5 μL of template DNA, and 0.2 μM of each primer. Two PCR assays were performed. The first PCR was conducted to amplify the *16S rRNA* gene for generic identification of mycobacteria. A segment (564 bp) was amplified using the primer set of 16MycF 5’-CGTGCTTAACACATGCAAGTCG-3’ and 16MycR 5’-GTGAGATTTCACGAACAACGC-3’ [33]. DNA was initially denatured at 95 °C for 2 min with subsequent 35 cycles of denaturation at 94 °C for 30 s, annealing at 52 °C for 30 s, and extension at 72 °C for 1 min with a final extension at 72 °C for 10 min [33]. Amplification was carried out in an Eppendorf thermocycler. The second PCR was performed to amplify the insertion sequence IS*901* (753 bp) for species identification of *M*. *avium*. Amplification was performed with the primer set IS*901*-F 5’-GAACGCTGCTCTAAGGACCTGTTGG-3’ and IS*901*-R 5’-GGAAGGGTGATTATCTGGCCTGC-3’ [34]. PCR mixture contained same reagent concentration as described previously. The reaction protocol was adopted from a previous study [35] but optimized to an initial denaturation at 95 °C for 3 min with subsequent 35 cycles of denaturation at 95 °C for 1 min, annealing at 60 °C for 40 s, extension at 72 °C for 35 s, and final extension at 72 °C for 10 min. *M*. *avium* subsp. *avium* (ATCC 15769) was used as positive control, and RNase-free water (Qiagen^®^) was used as negative control in each run of PCR assay. PCR products (5 μl) were analyzed by gel electrophoresis using 2% agarose. Agarose gel was stained with SYBR^®^ Safe DNA gel stain and viewed under UV light using AlphaImager^™^. Gel images were captured and labeled accordingly. Amplified fragments of the positive samples were sent for sequencing to the First Base Laboratories (Malaysia) and analyzed. Percentage of similarity to reference sequences was evaluated using NCBI-BLASTN tool [33].

### Statistical analysis

Data were entered in Microsoft Excel V.13, and SPSS v.22 was used for data analysis. Descriptive data were presented in frequency tables, percentages, and averages. Chi-square (χ^2^) test was used to compare decontamination procedures [22]. P-value < 0.05 was considered the level of significance.

## Results

### Spiked cultures

**Fig 3:**
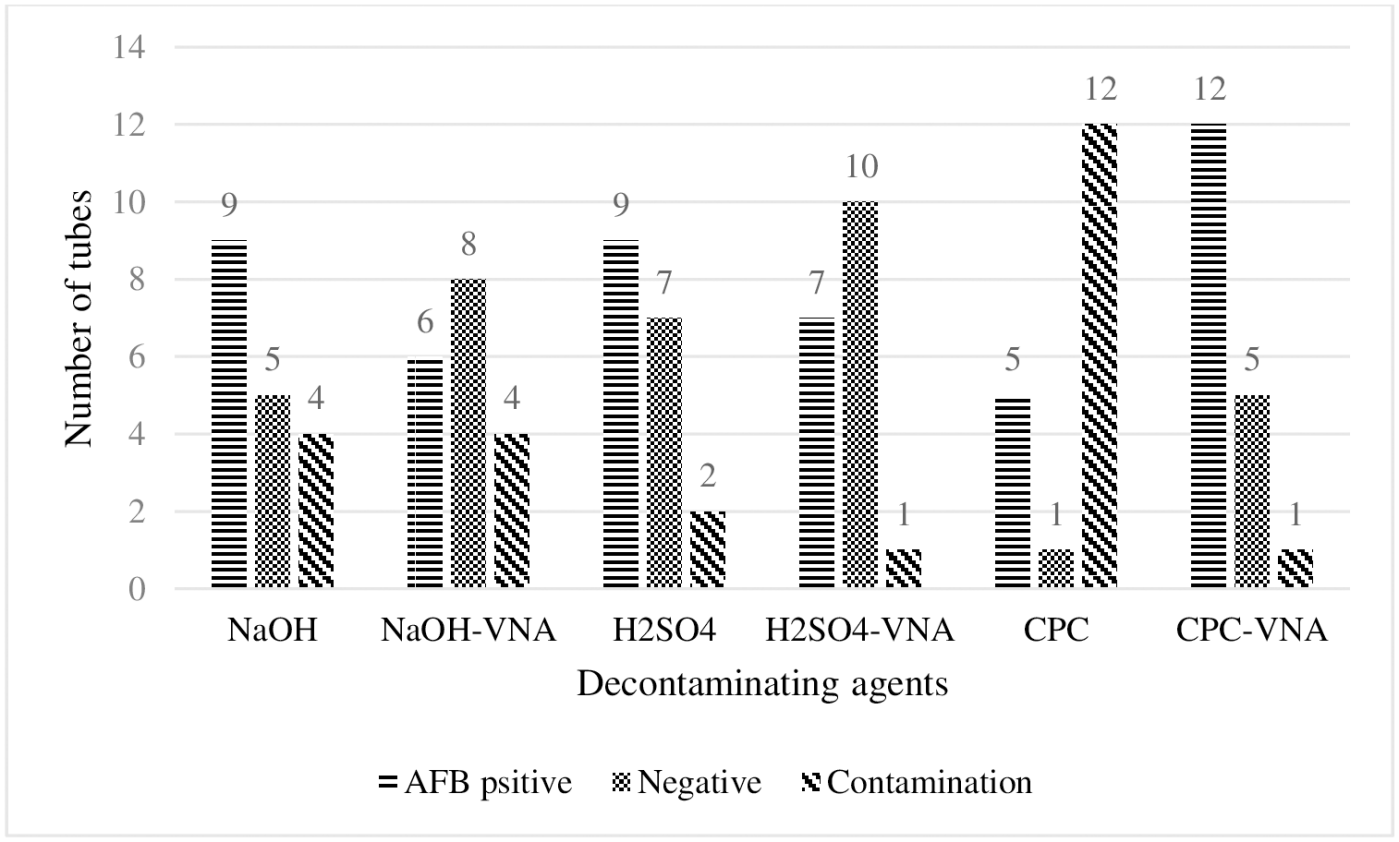
Number of tubes showing growth of *M*. *avium* subsp *avium* recovered from spiked fecal samples after six decontamination procedures. Bars show sum of AFB positive cultures, AFB negative cultures and contaminated cultures obtained from three inocula. When decontaminants were followed by VNA, a decrease in contamination rate was observed with all procedures, however the proportion of positive cultures increased with CPC 1%.

Bacterial growth on spiked cultures was categorized as confluent (uncountable) and countable numbers. The proportions of isolation of *M*. *avium* subsp. *avium* from spiked cultures (18 replicates) were as follows: NaOH, 9/18 (50%); NaOH-VNA, 6/18 (33.3%); H_2_SO_4_, 9/18 (50%); H_2_SO_4_-VNA, 7/18 (38.9%); CPC, 5/18 (27.8%); and CPC-VNA, 12/18 (66.7%) (Fig 3). *M*. *avium* subsp. *avium* was recovered significantly higher with CPC-VNA than those with NaOH-VNA and 1% CPC (27.8%; χ^2^ test, *p* = 0.01) (Table 1).

**Table 1:** P-values obtained by comparing six cultures procedures for the growth of *M*. *avium* subsp *avium* from spiked fecal cultures.

	NaOH	NaOH-VNA	H_2_SO_4_	H_2_SO_4_-VNA	CPC
NaOH					
NaOH-VNA	0.1				
H_2_SO_4_	1.0	0.1			
H_2_SO_4_-VNA	0.5	0.4	0.5		
CPC	0.1	1.0	0.1	0.4	
CPC-VNA	0.3	0.01*	0.3	0.09	0.01*

*Significant difference in the recovery of *M*. *avium* (27.8%; χ^2^ test *p* = 0.01).

CPC, with and without antibiotics, was less toxic to *M*. *avium* subsp. *avium* than those of NaOH and H_2_SO_4_. The toxicity of NaOH and H_2_SO_4_ was increased with the addition of VNA. This increase was indicated in the reduced mean colony count and proportion of positive cultures. However, first-time colonies appeared earlier with NaOH-VNA and H_2_SO_4_-VNA than those with NaOH and H_2_SO_4_ (Table 2). First colonies of *M*. *avium* subsp. *avium* with CPC-VNA appeared earlier (mean = 17.5 days) than that of any of the decontamination procedures. The average detection times to observe the first colonies of *M*. *avium* subsp. *avium* on L–J medium after the decontamination were 35.5 days for 4% NaOH and 12% H_2_SO_4_, 21–28 days for NaOH-VNA and H_2_SO_4_-VNA, and 21 days for CPC and CPC-VNA (Table 2).

**Table 2:** Mean colonies count and number of weeks for first appearance of growth of *M*. *avium* from spiked samples decontaminated using 6 procedures.

Decontaminant		NaOH	NaOH-VNA	H_2_SO_4_	H_2_SO_4_-VNA	CPC	CPC-VNA
Total number of replicates*		18	18	18	18	18	18
10^6^ CFU/ml	Positives n (%)	5 (83.3%)	5 (83.3%)	5 (83.3%)	6 (100%)	3 (50%)	6(100%)
	Contamination n (%)	1 (16.7%)	1 (16.7%)	1 (16.7%)	0 (0)	3 (50%)	0 (0)
	Negative n (%)	0 (0)	0 (0)	0 (0)	0 (0)	0 (0)	0 (0)
	Colony count (mean)	Confluent	Confluent	Confluent	51.6 ±15.7	Confluent	Confluent
10^4^ CFU/ml	Positives n (%)	4 (66.7%)	1(16.7%)	4 (66.6%)	1 (16.7%)	2 (33.3%)	6 (100%)
	Contaminated n (%)	2 (33.3%)	2(33.3%)	1 (16.7%)	0 (0)	4 (66.7%)	0 (0)
	Negative n (%)	0 (0)	3(50%)	1(16.7%)	5 (83.3%)	0 (0)	0 (0)
	Colony count (mean)	Confluent	35	11 ± 8.5	1	67.5 ± 45.9	13.6 ± 7
10^2^ CFU/ml	Positives n (%)	0 (0)	0 (0)	0 (0)	0 (0)	0 (0)	0 (0)
	Contaminated n (%)	1 (16.7%)	1 (16.7%)	0 (0)	1 (16.7%)	5 (83.3%)	1 (16.7%)
	Negative n (%)	5 (83.3%)	5 (83.3%)	6 (100%)	5 (83.3%)	1 (16.7%)	5 (83.3%)
First colony appearance (weeks)**	10^6^ CFU/ml	4^th^ week	2^nd^ week	4^th^ week	4^th^ week	2^nd^ week	2^nd^ week
	10^4^ CFU/ml	6^th^ week	4^th^ week	6^th^ week	4^th^ week	4^th^ week	4^th^ week

*Six replicates were spiked with each inoculum of *M*. *avium* subsp. *avium*.

**Colonies of *M*. *avium* were observed on the same day on all positive culture from 10^6^ and 10^4^ CFU/ml.

Positive control (10^5^) showed confluent growth of *M*. *avium* subsp. *avium*. All procedures except H_2_SO_4_-VNA showed confluent growth of *M*. *avium* subsp. *avium* from 10^6^ CFU/ml. The mean colony count of *M*. *avium* subsp. *avium* with H_2_SO_4_-VNA was 51±15.7. NaOH showed confluent growth from 10^4^ CFU/ml unlike other decontaminant methods (Table 2). More *M*. *avium* subsp. *avium* were recovered with NaOH than that with CPC-VNA, but the growth of *M*. *avium* subsp. *avium* with CPC-VNA was observed earlier than that with 4% NaOH (Table 2). Given that no *M*. *avium* subsp. *avium* was recovered after decontaminating the samples spiked with 10^2^ CFU/ml using any decontamination method, these results showed that the L–J culture media presented high sensitivity to recover *M*. *avium* subsp. *avium* from samples containing 10^6^ and 10^4^ CFU/ml. Similarly, despite that colony counts were not determined, these results allowed us to postulate that the bacterial kill rate for any decontamination method may be approximately 10^2^ CFU/ml (Table 2).

### Isolation of mycobacteria from fresh feces

**Fig 4:**
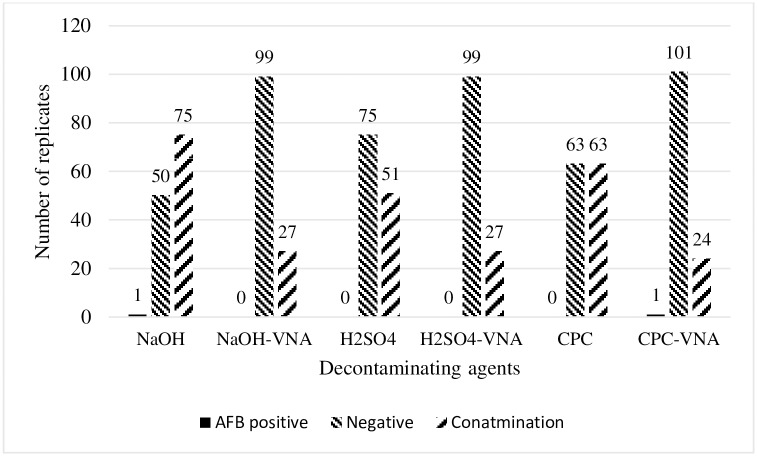
Number of tubes showing growth of mycobacteria recovered from field avian fecal samples using different decontamination procedures. Incorporation of VNA minimized the contamination of cultures in all procedures. However, VNA in NaOH significantly reduced the proportion of contaminated cultures (59.5%; χ^2^ test *p* = 0.0001) and CPC (50%; χ^2^ test *p* = 0.003).

**Fig 5:**
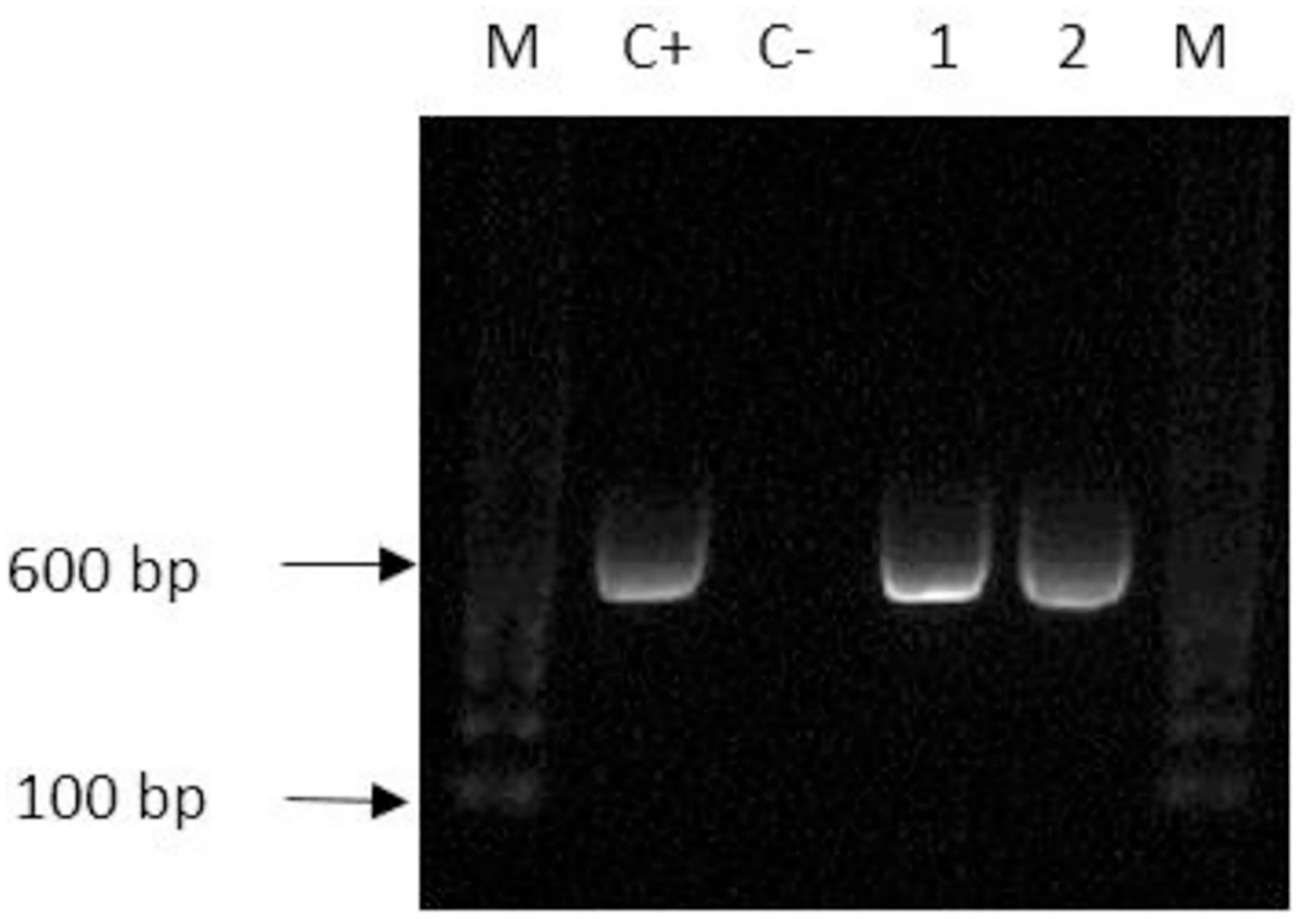
Gel picture of PCR (16S rRNA) of mycobacteria isolated from field samples. M, 100 bp molecular DNA marker; C+ positive control (*M*. *avium* subsp. *avium* ATCC 15769), C- negative control (sterile water); 1 and 2 (564 bp) positive samples from chicken.

Two mycobacterial isolates were obtained from fresh fecal cultures decontaminated with 4% NaOH and CPC-VNA (Fig 4), and first colonies were observed on the 12th and 15th days post-inoculation, respectively. Colonies were buffy in color, dry, rough, irregular in shape, and hard in consistency. Several parts of the media slant of both positive cultures showed blue discoloration, but the media were intact and in good condition until the 8th week of incubation. The isolates were confirmed first by AFB and subsequently by PCR. PCR amplified a 564 bp segment of *16S rRNA* gene (Fig 5). Both isolates were negative for IS*901*. Sequence analysis on *16S rRNA* gene revealed that the isolates were *Mycobacterium terrae* and *M*. *engbaekii*.

### Contamination of cultures

Culture contamination was defined as the complete overgrown of the media slant by nonacid fast organisms, as well as the breakage and liquefaction of at least one L–J slope. L–J slants were greenish in color prior to inoculation. Contamination of primary cultures appeared as mucoid white or yellow creamy colonies, thereby causing blue discoloration on L–J slants. Brown slimy colonies caused brown discoloration. Contaminating colonies covered the surface of the L–J slant. Contamination of primary culture was high during first week of incubation because most of the contaminating microorganisms multiply rapidly within 24 to 72 hrs. Severe contaminations caused breakdown on L–J slants, which resulted in liquefaction and finally collapsed of slant with the release of spoiled egg smell. The lowest proportion of contaminated cultures from the spiked fecal cultures was observed with CPC-VNA and H_2_SO_4_-VNA (1/18, 5.5%), H_2_SO_4_ (2/18, 11.1%), NaOH-VNA (4/18, 22.2%), NaOH (5/18, 27.8%), and CPC (12/18, 66.7%) in sequence. The proportion of contaminated cultures with CPC-VNA was significantly lower than that of 1% CPC (66.7%; χ^2^ test, *p*<0.001). Similarly, CPC-VNA showed that the lowest proportion of contamination on fresh fecal cultures was 24/126 (19%). The contamination rates with other procedures were 59.5%, 21.4%, 40.5%, 21.4%, and 50% for NaOH (75/126), NaOH-VNA (27/126), H_2_SO_4_ (51/126), H_2_SO_4_-VNA (27/126), and CPC (63/126), respectively (Fig 4). Contamination on fresh fecal cultures with CPC-VNA (19%) was significantly lower than those with NaOH (*p*<0.0001), H_2_SO_4_ (*p*<0.05) and CPC (*p*<0.003). However, the difference in contamination on fresh fecal cultures with CPC-VNA was insignificant compared with those with NaOH-VNA and H_2_SO_4_-VNA (*p*<0.786).

## Discussion

This study showed that the decontamination of fecal samples to isolate *M*. *avium* subsp. *avium* on culture media can be performed using 4% NaOH, 12% H_2_SO_4_, and 1% CPC. These three decontaminant agents showed no significant difference in the recovery of *M*. *avium* subsp. *avium* from feces. Nevertheless, the proportion of contaminated cultures was high for samples decontaminated with CPC.

The bacteriostatic effects of these three chemicals on mycobacteria and other bacteria can vary in accordance to their concentrations. Corner et al [21]reported that the increase in NaOH concentration from 0.1% and 2% can reduce the viability of *M*. *bovis* from 13% to 60% compared with the 20% reduction with CPC when the concentration increases from 0.375% to 1.5% [21]. Kamerbeek et al. reported that 4% NaOH can kill more than 60% of the bacilli [36], and Peres et al. showed that an increase in NaOH concentration from 1% to 1.25% reduces the sensitivity of culture to 52% [37]. Similarly, Chatterjee et al. reported that the increase in NaOH concentration from 2% to 4% can reduce the contamination of culture media and the number of positive cultures [38]. Furthermore, malachite green, which is the selective antifungal agent in L–J, shows inhibitory effects on the growth of different mycobacterial species, including MAC. In a previous study, Brooks et al. found that certain MAC isolates are significantly susceptible to malachite green [39]. It is noteworthy to mention that more literature about comparison of decontamination procedures have focused primary isolation of *M*. *tuberculosis* and *M*. *bovis*.

The toxic effects of acids and alkalis depend on H^+^ and hydroxyl ion concentrations. Hydrogen ions (H^+^) and change in pH in the cytoplasm destroy peptide bonds in the nucleic acid and precipitate proteins [40]. Additionally, OH^−^ ions cause lipid saponification in cell membrane, which leads to cellular destruction [40]. Moreover, pH of more than 10 disorganizes the structure of peptidoglycan and causes the hydrolysis of nucleotides [39,41]. Mycobacteria are resistant to chemicals and antimicrobials due to the presence of mycolic acid, which makes the outermost layer of the cell wall [42]. Mycolic acid consists of long chain fatty acids, which make a waxy and non-fluid barrier [42]. NaOH is more toxic to fast growing mycobacteria compared to *M*. *tuberculosis* or MAC [43]. H_2_SO_4_ isolates NTM more effectively than that of *Mycobacterium tuberculosis* [44]. To ensure that the decontamination of sample with NaOH and H_2_SO_4_ will affect mycobacterial growth, the decontamination time and the neutralization of NaOH and H_2_SO_4_ activity should be controlled properly [22]. CPC is a cationic quaternary ammonium compound that is relatively hydrophobic, and it destabilizes the lipid bilayer in the cell wall by replacing the divalent calcium ion (Ca^2+^) [45]. Progressive CPC adsorption to acidic phospholipids decreases the fluidity of the lipid bilayer and creates hydrophobic channels in the membrane. Finally, protein functions are hindered with cell lysis [45]. Unlike NaOH and H_2_SO_4_, CPC activity requires no neutralization, and long exposure to CPC exerts no significant effect on mycobacterial growth [29]. Moreover, treatment of feces with 2% NaCl effectively liquefies the fecal matrix which helps in proper decontamination with 1% CPC [46]

With regard to the use of VNA, the present study evaluated alternate decontamination methods to obtain many positive cultures with minimum contamination. The VNA antibiotics reduced the contamination rates after the decontamination with NaOH, H_2_SO_4_, and CPC. Nonetheless, the effects of VNA on samples previously treated with CPC were considerably important because it reduced the contaminated cultures and increased the number of positive cultures compared with those of NaOH-VNA and H_2_SO_4_-VNA. Although NaOH-VNA and H_2_SO_4_-VNA exerted negative effects on the final recoveries of positive cultures, these combinations presented no effects on the time to detect the first colonies on the culture. Early appearance of colonies on culture media may be explained by that *M*. *avium* subsp. *avium* may grow rapidly because of low competition for nutrients after the complete removal of contaminating organisms. Our results were similar to those of previous study, in which the authors compared six different decontaminating procedures and found no significant difference in the recovery of *M*. *avium* and *M*. *chelonae* from spiked water samples [24]. However, the recovery of these mycobacteria with 0.1% CPC was higher than those with 4% NaOH, 4% H_2_SO_4_, 5% oxalic acid, 2% NaOH and *N*-acetyl L-cysteine and sodium dodecyl sulphate (3%) and NaOH (1%). Notably, the methodology in the previous study was different from that in the current study. They decontaminated water samples with 4% NaOH, 4% H_2_SO_4_, and 0.1% CPC and cultured on Middlebrook 7H11 agar supplemented with oleic acid, albumin, dextrose, and catalase and antibiotics (PANTA). Contrary to L-J, colonies of mycobacteria are easily visible and seen earlier on Middlebrook 7H11 agar than those on egg-based L–J media. Unlike L-J, contamination does not cause liquefaction of agar, but it masks the growth of mycobacteria [47].

In the present study, the use of CPC and VNA had a better performance to recovery *M*. *avium* from fecal samples spiked with 10^6^ and 10^4^ CFU/ml than 10^2^ CFU/ml. Despite that this low sensitivity to detect *M*. *avium* was also reported by Reddacliff et al. [29], it is thought that some factors/variables e.g., dividing each spiked fecal preparation into three aliquots reduced the number of bacilli by threefold. Furthermore, the selection of 100 μL as the final inoculum for culture further reduced the number of bacilli could have an impact the results of this study.

Among some limitations of this study, type of the samples used for culture and low number of samples from pet birds are included. A large number of village chicken samples were used because pet birds are rarely available in the area. Village chickens were free to roam in fenced areas. The sample collection procedure adopted in the current study was inapplicable to these birds. Most of the feces from village chickens were collected from the ground surface. Therefore, culture contamination will be overestimated. Small sample size for spike experiment, low number of bacilli in each aliquot of inocula, and culture contamination were other limitations of this study. Inclusion of negative control was not made as the tube containing *M*. *avium* (Positive control) and feces was evaluated for positive and contamination results. Another limitation was the determination of *M*. *avium* subsp. *avium* inoculated into each spiked sample in CFU/ml. Given that *M*. *avium* subsp. *avium* can form clumps, the inoculum volume (100 μl) can exert a negative effect on the number of bacilli inoculated on the culture media. The implementation of CFU method in determining the concentration of bacilli spiked into samples and recovered in each step of the procedure (e.g., post-exposure of bacilli to decontaminant agents, post-centrifugation, post-resuspension of sediment, and recovery of bacilli on culture media) should be further investigated. Knowing the CFU per milliliter after each process, we might accurately conclude if the decontamination with chemical agents or antibiotic mixture is more toxic against *M*. *avium*. Similarly, the sensitivity of L–J media can be determined accurately because L–J media can isolate approximately 10 bacilli [47]. With regard to the number of fresh samples used, further studies should include a large number of chicken and pet bird samples to determine the prevalence of *M*. *avium* or other mycobacteria in these birds.

In conclusion, the decontamination of chicken fecal samples by using CPC 1% with subsequent incubation with VNA for 24 h is a suitable method to isolate *M*. *avium* and reduce the contamination rate on solid media. This method is simple, and it increases the recovery of *M*. *avium* on solid culture media from bird fecal samples.

## Supporting information

S1 File Supporting document-Data.xlsp: Data of spiked samples comparing six decontamination procedures. (XLSX)
